# Evaluation of the composition and in vitro antimicrobial, antioxidant, and anti-inflammatory activities of Cilantro (*Coriandrum sativum* L. leaves) cultivated in Saudi Arabia (Al-Kharj)

**DOI:** 10.1016/j.sjbs.2021.03.011

**Published:** 2021-03-13

**Authors:** Ahmed I. Foudah, Mohammad H. Alqarni, Aftab Alam, Mohammad Ayman Salkini, Elmutasim O. Ibnouf Ahmed, Hasan S. Yusufoglu

**Affiliations:** a Department of Pharmacognosy, College of Pharmacy, Prince Sattam Bin Abdulaziz University, Al Kharj 11942, Saudi Arabia; b Department of Pharmaceutics, College of Pharmacy, Prince Sattam Bin Abdulaziz University, Al Kharj 11942, Saudi Arabia; c Omdurman Islamic University/Faculty of Medical Laboratory Sciences/Department of Medical Microbiology, Sudan

**Keywords:** Cilantro, Saudi Arabia, Essential oil, Composition, Antioxidant, Antimicrobial, Anti-inflammatory

## Abstract

**Background:**

In the present study, we explored the composition of Cilantro (*Coriandrum sativum* L. leaves) essential oil (CEO) cultivated in Saudi Arabia (Al-Kharj) and explored its antioxidant, antimicrobial, and anti-inflammatory effects in vitro.

**Methods:**

Gas chromatography-mass spectroscopy was used to detect the CEO composition. The 2, 2-diphenyl-1-picrylhydrazyl (DPPH)-induced free radical and ferric chloride scavenging methods were used to determine the antioxidant activity. Antimicrobial activity was investigated using the well diffusion method. Anti-inflammatory activity was evaluated using egg albumin and trypsin-induced inflammation methods.

**Results:**

Forty-six compounds representing 90.17% of the total aroma were identified in the CEO; the major constituents were found to be 1-decanol (17.85%), decanal (11.04%), *trans*-2-dodecen-1-ol (7.87%), menthone (6.71%), 2-decen-1-ol, *trans*- (5.44%), dodecanal (4.76%), *trans*-tetradec-2-enal (3.14%), sedanolide (3.02), and thymol (3.01%). DPPH-induced free radical and ferric chloride scavenging assays demonstrated low antioxidant effects of CEO, and the antioxidant activity was observed at a high CEO concentration. The antimicrobial activity of CEO was assessed against 5 microorganisms (bacteria and fungi) by using well diffusion methods; CEO was found to possess excellent antimicrobial activity against all microorganisms, except *Escherichia coli*. Moreover, CEO demonstrated strong anti-inflammatory activity against egg albumin- and trypsin-induced inflammation.

**Conclusion:**

The essential oil extracted from *C. sativum* chemotype grown in Al-Kharj region of Saudi Arabia possesses low antioxidant potential, superior antimicrobial activity, and outstanding anti-inflammatory effects.

## 1 Introduction

The development of new antimicrobial, antioxidant, and anti-inflammatory agents is one of the challenging fields of research because of antibiotic resistance and toxicity issues of synthetic antioxidants and anti-inflammatory agents. Resistance to antibiotic agents varies across regions or individuals. The available synthetic antioxidants have been reported to cause adverse health-related issues, such as cancer, whereas the available anti-inflammatory agents have been reported to cause severe gastrointestinal, cardiac, liver, and kidney toxicities (Cheesman et al., 2017, Bindu et al., 2020). To minimise the adverse side effects of the available drugs and to enhance their therapeutic effects, the compounds originating from natural sources may be used as an alternative to the synthetic compounds (Lobo et al., 2010).

Leaves of *Coriandrum sativum* L. (annual herb; Family Apiaceae), often known as 'Cilantro', are edible. The plants are widely distributed across the world and are cultivated predominantly in different region of Saudi Arabia. It was originated in the Italian parts of the Mediterranean region, but nowadays cultivated and consumed worldwide (Sahib et al., 2013). Aerial parts of the plant are traditionally used as food materials and in the treatment of cancer, diabetes, and liver and stomach disorders (Hwang et al., 2014). Essential oil found in leaves and seeds of this plant is nontoxic to humans, and thus, it is used as a preservative and flavouring agent in food preparation, as a therapeutic agent in pharmaceutical productions, and as fragrance in perfumes (Mandal and Mandal, 2015). Several scientific investigations have suggested that ingestion of coriander leaves is associated with many health benefits because of the presence of pharmacologically active metabolites in their essential oil (Laribi et al., 2015, Jawad et al., 2018). Seed oil comprises mainly linalool, pinene, cymene, borneol, phellandrene, and geraniol; however, the chemical constituents of leaf essential oil was reported to be completely different from those of the seed essential oil (Önder and El-Shemy, 2018). Essential oil of seeds mainly contains linalool, whereas that of leaves contains decanal, decanol, cyclodecane, and dodecena (Mandal and Mandal, 2015).

Currently, many consumers prefer to use antioxidant, antimicrobial, and anti-inflammatory additive foods of natural origin. Essential oils obtained from plants and their components have been shown to retain the properties of those obtained from natural origin. However, no literature is available on the chemical composition and biological activities of the essential oil found in *C. sativum* chemotype cultivated in Al-Kharj region of Saudi Arabia. Therefore, the aim of this study was to explore the composition of essential oil found in the leaves of *C. sativum* chemotype grown in Al-Kharj region of Saudi Arabia and evaluate the antimicrobial, antioxidant, and anti-inflammatory properties.

## 2 Materials and methods

### 2.1 Plant materials and isolation of essential oil

Cilantro (leaves of *C. sativum*) was collected from the local farm of Al-Kharj, Riyadh Province of Saudi Arabia, in July 2020. The plant was identified by Dr. Osman Al-Makki, College of Pharmacy, Prince Sattam bin Abdulaziz University (PSAU) and a voucher specimen (CS.012-2020) was deposited in the herbarium laboratory (Pharmacognosy), College of Pharmacy, PSAU.

### 2.2 Cilantro essential oil

Powder of dried Cilantro (600 g) was hydro distilled for 4 h by using a Clevenger-type apparatus to isolate the essential oil. Hydro-distilled essential oil was separated from water, dried over sodium sulphate (anhydrous), and stored in amber colour glass vials at 4 °C in refrigerator for the further evaluation

### 2.3 Gas chromatography-mass spectrometry analysis

Gas chromatography-mass spectrometry (GC-MS) analysis of cilantro essential oil (CEO) was performed on a GC coupled to MS (Agilent 7890B GC and Agilent 5977B MSD respectively) and fitted with a HP-5 MS capillary column (containing fused silica and having inner diameter of 0.25 mm, length of 30 m, and film thickness of 0.25 µm). The temperature of oven was kept at 70 °C for 5 min, and then it was increased to 290 °C at a speed of 10 °C/min, maintained at 290 °C for 5 min. The inlet temperature was 280 °C, and transfer line temperature was 290 °C. Helium (>99.999%) was used as the carrier gas at a flow rate of 1 mL/min. Aliquots of diluted oils (1 mL of 1 ppm in methanol) were injected. The injection method was analysed in the split-less mode. The scanning mass range was 30–600 *m*/*z*, and the ionisation energy of the detector was 70 eV. The components were identified by comparing their retention times with the retention times of authentic standards, and mass spectra with National Institute of Standards and Technology (NIST 2017). The analysis and processing of the results were controlled using MASSHUNTER software.

### 2.4 Determination of antioxidant activities

Antioxidant activity of CEO was evaluated using 2, 2-diphenyl-1-picrylhydrazyl (DPPH) radical scavenging assay described by Alqarni et al. (Alqarni et al., 2019) and ferric chloride reducing method described by Alam et al. (Alam et al., 2019), with slight modifications.

In the DPPH free radical scavenging assay, 0.5 mL of various CEO dilutions (0.25–5 mg/mL) was added to 2 mL of DPPH (0.004%, methanol solution). The reaction mixture was vigorously shaken and kept in dark for 30 min at room temperature. Ascorbic acid was used as the standard. The absorbance of the reaction mixture was measured at 517 nm by using a ultraviolet–visible (UV–Vis) spectrophotometer. The percentage of DPPH free radical scavenging (FRS) potential was calculated using the following equation:

FRS (%) = 100 (1-A_(test)_ /A_(control)_); where A(test) represents the test sample.

In the ferric chloride reducing assay, 1 mL of CEO (0.25–5 mg/mL), 2.5 mL of phosphate-buffer (pH 6.6; 0.2 M), and 2.5 mL of potassium ferricyanide (K_3_[Fe (CN)_6_; 1%) were mixed. The combination was incubated for 20 min at 50 °C; the reaction was terminated by adding 2.5 mL of trichloroacetic acid (TCA, 10%), and the reaction mixture was centrifuged for 10 min at 6000 r.m.p. The supernatant layer (2.5 mL), 2.5 mL of distilled-water, and 2.5 mL of 0.1% FeCl_3_ solution were mixed. Ascorbic acid was used as the standard. The absorbance was measured at 700 nm by using a UV–Vis spectrophotometer. Increase in the absorbance of the reaction mixture indicated the antioxidant potential.

### 2.5 Determination of antimicrobial activities

Antimicrobial activities of CEO were assessed against gram-positive bacteria (*Bacillus subtilis ATCC 11774*, *Staphylococcus aureus ATCC 25923*), gram-negative bacteria (*Escherichia coli ATCC 11229, Klebsiella pneumonia NCTC 9633*), as well as a pathogenic fungus (*Candida albicans ATCC 10231)*. All the strains were obtained from the College of Pharmacy, Prince Sattam bin Abdulaziz University, Al-Kharj. The obtained bacteria were subcultured for 24 h onto Mueller-Hinton agar (M−H agar, Hi Media laboratories), and the fungal strain *C. albicans* was cultured on Sabouraud-dextrose agar (S-D agar, Hi Media laboratories) for 5 days at 37 °C. Colonies from subcultured plates were grown in M−H broth and S-D broth to match the 0.5 McFarland turbidity standard equivalent to 1.5 × 10^8^ CFU/mL. M−H agar and S-D agar were used for antibacterial and antifungal assays, respectively, and the agar-well diffusion method (Alkahtani et al., 2020) was used.

In this method, 15 mL of molten-cooled agar was poured into a petri dish, and 0.1 mL of inoculum of each test microorganism was spread onto sterile agar; agar was allowed to solidify. A 6-mm diameter well was punched in solidified agar by using a sterile cork-borer and filled with 100 μL of 5% (w/v) CEO (in DMSO). The plates were placed in a refrigerator for 30 min to allow proper diffusion of oil into agar and incubated for 24 h at 37 °C. DMSO (5% v/v) was used as the negative control, and each test was performed in triplicate. A clear zone of inhibition around the well was observed, and the diameter of inhibition was measured in millimetres. The CEO exhibited antimicrobial activity at 5% (w/v) DMSO, and thus, this concentration was further manipulated to explore its minimum inhibitory concentration (MIC) by using the diffusion method (Gonelimali et al., 2018). Different CEO dilutions (4%, 2%, 1%, 0.5%, and 0.25%) were prepared using the two-fold serial dilution method. Subsequently, 0.1 mL of each inoculum was spread onto the sterile agar petri dishes, and 5 wells were punched on each plate; 100 μL of 4%, 2%, 1%, 0.5%, and 0.25% CEO was transferred separately to each well. Plates were incubated at 37 °C for 18 h after placing them in a refrigerator for 30 min; each test was performed in triplicate. The MIC was calculated as the lowest concentration at which the growth of individual microorganisms was inhibited.

To explore the bactericidal effects of CEO, the time–kill assay reported by Li et al. was used (Li et al., 2019). Microorganisms were incubated for 8 h in nutrient broth agar at 37 °C; the suspension was centrifuged and resuspended in saline to nearly 10^6^ CFU/mL. The suspension of microorganism was treated with nutrient broth medium holding different CEO dilutions (5% DMSO) with variable MIC. The inoculums were mixed and cultured at 37 °C; the samples were taken at selected time intervals (0, 4, 8, 12, 18, and 24 h) from the culture, diluted with saline, and cultured on nutrient broth medium. The colony-forming unit (CFU) was calculated after incubating the plates for 24 h at 37 °C. A time–kill assay graph between log CFU/mL and time was plotted.

### 2.6 In vitro anti-inflammatory activity assay

The anti-inflammatory activity of CEO was evaluated using albumin denaturation (AD) method described by Alam and Singh, (Alam and Singh, 2020) and proteinase inhibitory method described by Gunathilake et al. (Gunathilake et al., 2018), with slight modifications.

In the egg albumin method, the reaction mixture, 2.8 mL of phosphate-buffered saline (PBS, pH 6.4), 0.2 mL of fresh hen egg albumin, 2 mL of different CEO dilution (5, 10, 50, 200, and 1000 μg/mL), and Ibuprofen (standard drug) were added in separate test tubes. The reaction mixtures were incubated for 15 min at 37 ± 2 °C, subjected to heating in oven at 70 °C for 10 min, and then allowed to cool; the absorbance of the reaction mixture was measured at 660 nm by using a UV–Vis spectrophotometer; PBS solution was used as the blank. The experiment was repeated thrice, and the percentage inhibition of AD was measured using the following equation:

% inhibition (AD) = 100 (1 – A sample/A control), where A sample represents the test sample and A control represents the control

In the proteinase inhibitory assay, 0.06 mg trypsin, 1 mL of tris-HCl buffer (20 mM), 1 mL of different CEO dilution (5, 10, 50, and 200 μg/mL), and Ibuprofen (standard drug) were added in separate test tubes and mixed thoroughly. The reaction mixtures were incubated at 37 °C for 15 min. The mixtures were again incubated at 37 °C for 20 min after adding 1 mL of casein (0.8% w/v). Then, 2 mL of 70% per chloric acid was added to each mixture, and the mixture was centrifuged at 3000 rpm for 5 min. Supernatant of the centrifuged samples was separated, and absorbance of each sample was measured at 210 nm. The mixture containing all the reagents except the sample and standard solution was used as the control. The experiment was repeated thrice, and the percentage of protein denaturation was calculated using the following equation:

% inhibition (protein denaturation) = 100 (1 − A sample/A control), where A sample represents the test sample and A control represents the control.

### 2.7 Statistical analysis

The antimicrobial inhibition percentage was presented as the average of the three consecutive experiment, whereas the antioxidant and anti-inflammatory inhibition percentages were reported as the mean ± standard deviation. The IC50 value (50% inhibition) of anti-inflammatory inhibition was calculated using regression equations obtained from the graph between percentage (%) inhibition and concentration. All values were calculated using GraphPad prism software (version 9.0.0; 121).

## 3 Results

### 3.1 Gas chromatography-mass spectrometry analysis

**Fig. 1:**
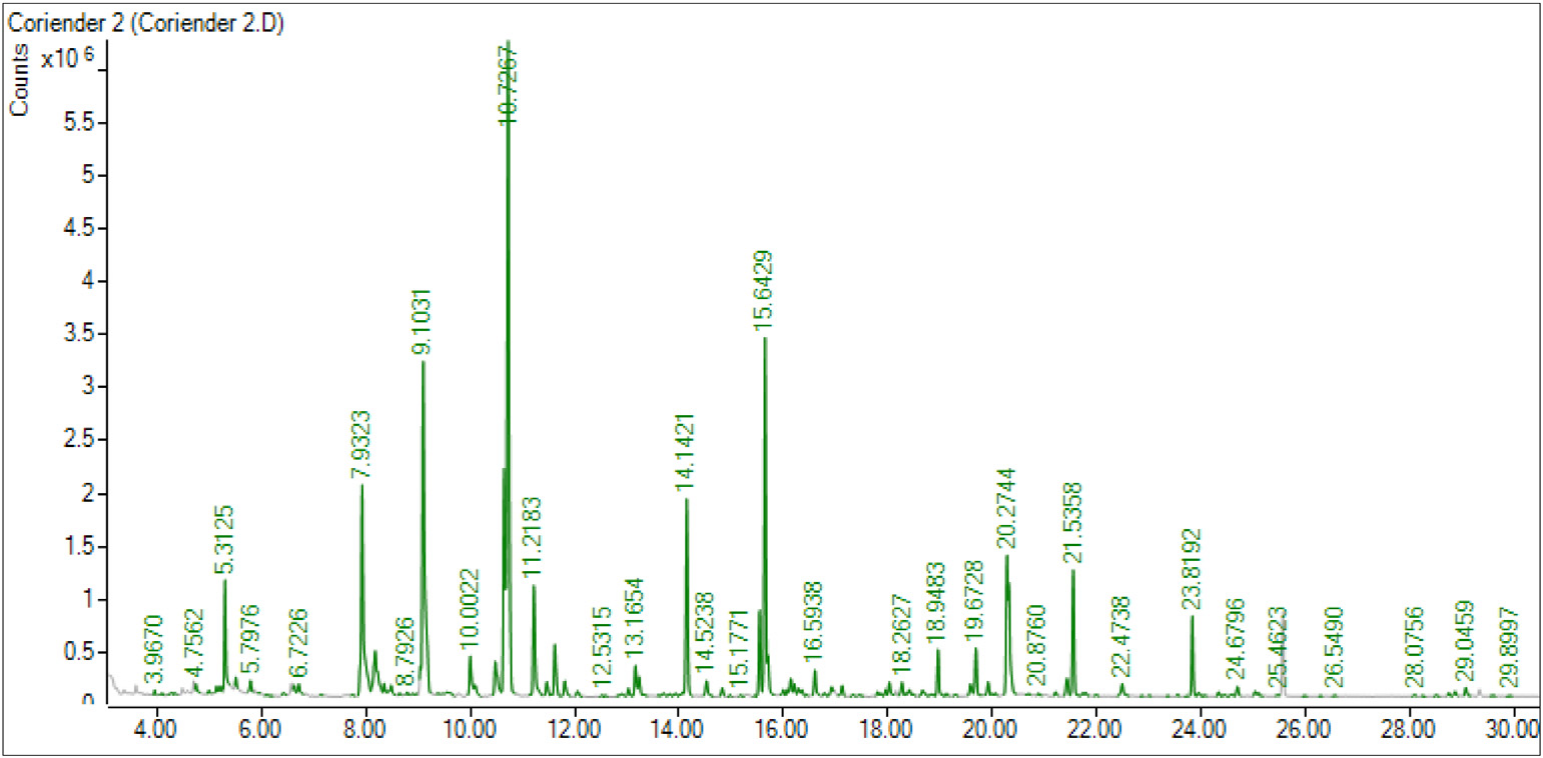
GC–MS chromatogram of CEO.

We identified 46 volatile compounds, namely monoterpene (17.47%), sesquiterpenes (2.96%), diterpenes (2.28%), phthalates (4.24%), and non-terpenes (63.23%), in the CEO by using GC-MS. Fig. 1 shows the detailed compositions obtained in the chromatogram, and Table 1 represents the percentage of each component. The major constituents were found to be 1-decanol (17.85%), decanal (11.04%), *trans*-2-dodecen-1-ol (7.87%), menthone (6.71%), 2-decen-1-ol, *trans*- (5.44%), dodecanal (4.76%), *trans*-tetradec-2-enal (3.14%), sedanolide (3.02), and thymol (3.01%).

**Table 1:** Chemical composition CEO.

Name	RT	Area %
**Monoterpene**		
Eucalyptol	5.3125	2.25
γ-Terpinene	5.7976	0.28
Linalool	6.6321	0.23
Menthone	7.9323	6.71
*trans*-p-Menthan-3-one	8.1845	2.46
p-Menthan-3-ol	8.3527	0.29
p-Menth-1-en-4-ol	8.4886	0.35
Pulegone	10.0022	1.15
Carvone	10.0992	0.36
Thymol	11.2183	3.01
Carvacrol	11.4641	0.38
%		17.47%

**Sesquiterpenes**		
Caryophyllene	14.5238	0.47
β-Selinene	16.1345	0.42
Dihydroagarofuran	16.2056	0.27
Kessane	17.1177	0.29
Spathulenol	18.2627	0.37
τ-Cadinol	19.6728	1.14
%		2.96%
**Diterpenes**		
Isophytol	18.6573	0.22
Neophytadiene	23.8192	1.61
*cis*-phytol	24.6796	0.24
Phytol	29.0459	0.21
%		2.28%

**Phthalate**		
Senkyunolide	21.4194	0.51
Sedanolide	21.5358	3.02
Hexahydro-3-butylphthalide	19.5693	0.36
3-Butylisobenzofuran-1(3H)-one	19.9122	0.35
%		4.24%

**Non-terpenes (Fatty acid, alcohol and aldehyde)**		
Nonanal	6.7226	0.31
Decanal	9.1031	11.04
*trans*-2-Decenal	10.4809	1.42
*trans*-2-Decen-1-ol	10.6491	5.44
1-Decanol	10.7267	17.85
Undecanal	11.6194	1.40
Phenol, 5-ethenyl-2-methoxy-	11.8134	0.45
*trans*-2-Undecen-1-ol	13.1654	0.78
1-Undecanol	13.2301	0.52
Dodecanal	14.1421	4.76
*trans*-2-Dodecenal	15.5394	1.94
*trans*-2-Dodecen-1-ol	15.6429	7.87
1-Dodecanol	15.7011	0.90
Tridecanal	16.5938	0.65
Undecanoic acid, 10-methyl-, methyl ester	16.9172	0.28
*trans*-2-Tridecen-1-ol	18.0233	0.39
Tetradecanal	18.9483	1.08
*trans*-Tetradec-2-enal	20.2744	3.14
*trans*-2-Dodecen-1-ol	20.3068	2.58
*trans-*Hexadec-2-enal	22.4738	0.42
%		63.23%
Monoterpene		90.17%

Notes: Compounds are listed in order of their elution from a HP-5MS column; RT (retention time): on a HP-5MS column.

### 3.2 Determination of antioxidant activities

**Fig. 2:**
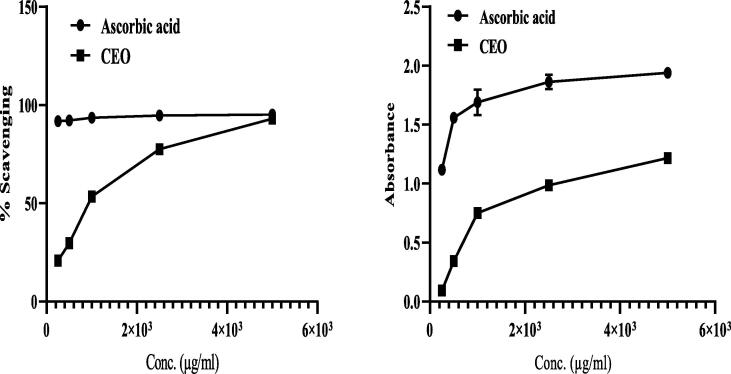
Antioxidant data of CEO given as (A) percentage (%) of scavenging DPPH induced free radical and (B) absorption of ferric chloride reducing assay, results were reported as Mean ± SD of the three experiments.

Antioxidant activities of CEO were determined using two assays, namely DPPH assay and ferric chloride reducing assay; the results are presented in Fig. 2.

In the 1,1-diphenyl-2-picrylhydrazyl method, the stable free radical (FR), DPPH (colour: deep violet), reacts with the antioxidant compound and is converted to 1,1-diphenyl-2-picrylhydrazine (discoloration), which indicates the FRS activity of the antioxidant compound/extracts. In the DPPH assay, CEO was found to scavenge the DPPH-induced free radical by 93.12% ± 0.52% at 5 mg/mL (Fig. 2), whereas the standard ascorbic acid was found to scavenge the DPPH-induced free radical by 93.58 ± 0.22% at 1 mg/mL.

### 3.3 Antimicrobial activity

Antimicrobial activity of CEO was evaluated in terms of the inhibition zone (IZ) and MIC; Table 2 presents the results in detail.

**Table 2:** Antimicrobial activity of CEO*.*

Microorganism	CEO (ZI; mm, MIC %)			
	0.5%	1%	2%	MIC
*S. aureus (ATCC26923)*	17	21.66	27.66	0.05
*S. subtilis (ATCC11774)*	18	22.66	32	0.0125
*E. coli (ATCC11229)*	–	–	–	–
*K. pneumoniae (NCTC9633)*	17.66	23.33	24.66	0.0125
*C. albicans (ATCC10231)*	17.33	18.66	22	0.0125

The IZ varied slightly with different CEO dilutions and microorganisms used in the assay. CEO was found to be inactive against *E. coli,* and therefore, the MIC for *E. coli* was not assessed. The MIC value ranged between 0.05% and 0.0125% against all the tested microorganism.

**Fig. 3:**
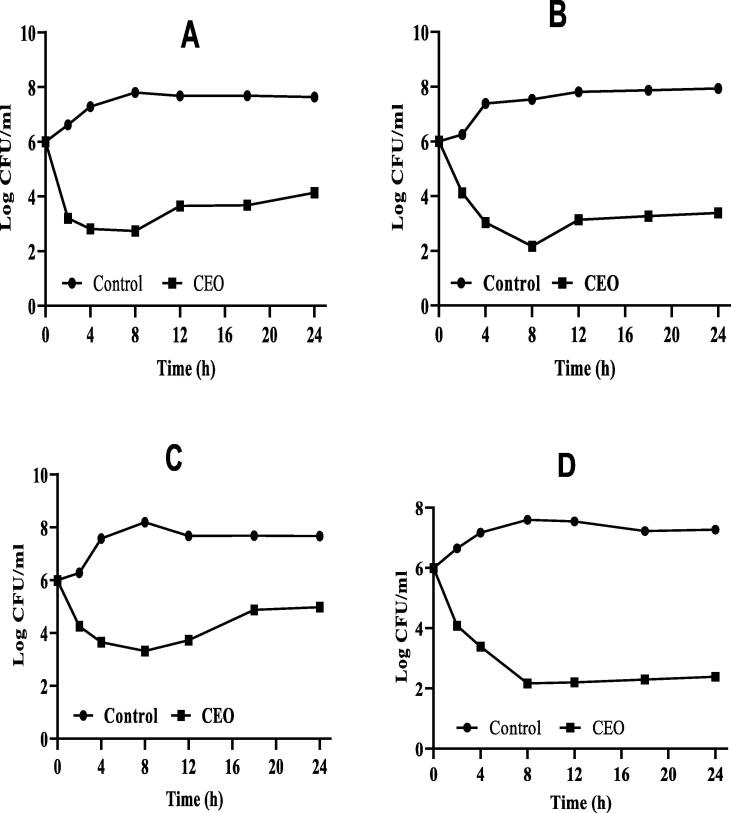
Time-kill analysis of (A) *S. aureus;* (B) *S. subtilis;* (C) *K. pneumonia*; and (D) *C. albicans.*

Time–kill assays of CEO were executed to explore the cell viability (kill time) against the tested microorganisms, and the results are expressed as a logarithm of viable counts (Fig. 3A–D). The log_10_ CFU/mL of the untreated *S. aureus* increased from 6 to 7.80 and reached a static phase after 8 h. However, the log_10_ CFU/mL of CEO-treated *S. aureus* sharply decreased in the first 8 h and increased steadily to approximately 4.14 log_10_ CFU/mL (Fig. 3A). The log_10_ CFU/mL of untreated *S. subtilis* increased steadily from 6 to 7.94 and transited into a static phase after 2 h; in CEO-treated *S. subtilis* the log_10_ CFU/mL decreased sharply in the first 4 h and increased steadily to approximately 3.39 log_10_ CFU/mL (Fig. 3B). Similarly, the log_10_ CFU/mL of untreated *K. pneumonia* increased from 6 to 8.2 and turned into a static phase after 8 h, whereas in CEO-treated *K. pneumonia,* log_10_ CFU/mL decreased sharply in the first 4 h and gradually reached approximately 4.8 log_10_ CFU/mL (Fig. 3C). The log_10_ CFU/mL of the untreated *C. albicans* culture increased in the first 8 h and was maintained steadily at approximately 7.68 log_10_ CFU/mL, whereas that of CEO-treated *C. albicans* culture decreased sharply in the first 8 h and increased steadily to approximately 4.8 log_10_ CFU/mL (Fig. 3D). Time–kill assays indicated that CEO had killing effect on the growth of *S. aureus, S. subtilis, K. pneumonia*, and *C. albicans*.

### 3.4 Anti-inflammatory activity

**Fig. 4:**
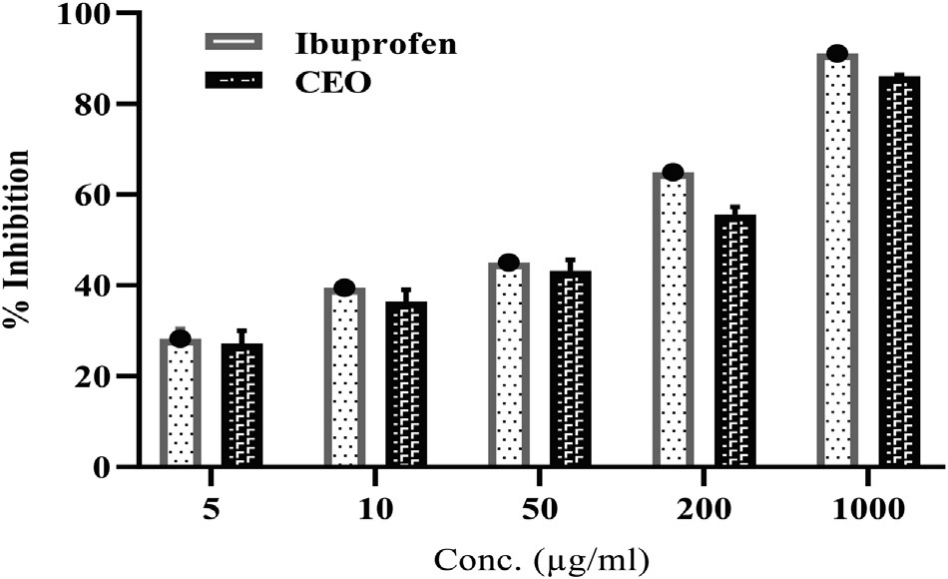
BSA inhibitory assay of CEO and Ibuprofen. Reported as mean ± SD of triplets.

**Fig. 5:**
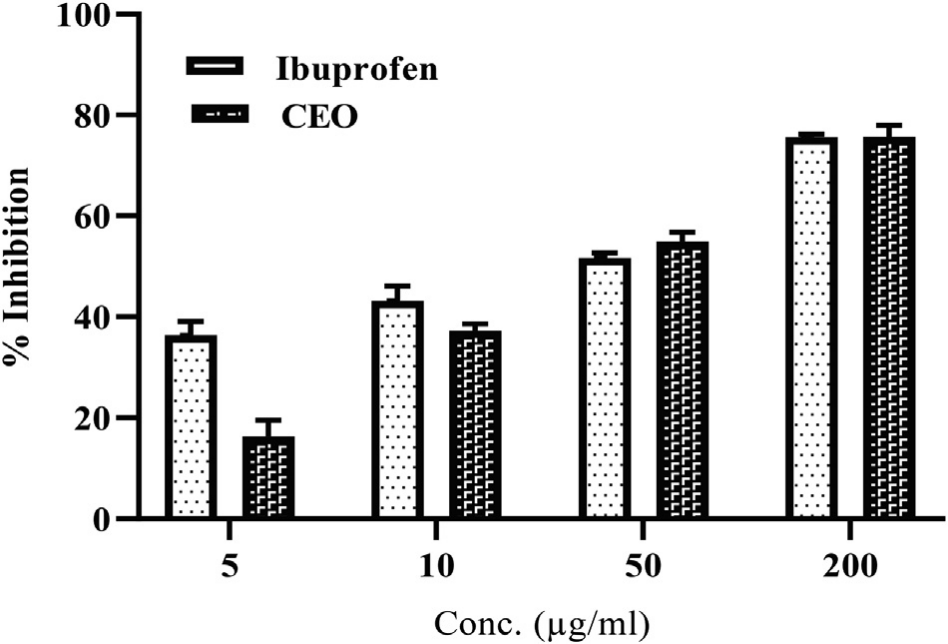
Proteinase inhibitory assay of CEO and Ibuprofen. Reported as mean ± SD of triplets.

The anti-inflammatory activity of CEO was determined using two assays, namely egg albumin assay and proteinase inhibitory assay; Fig. 4, Fig. 5 presents the results of both assays, respectively.

In the egg albumin assay, the percentage inhibition of egg albumin-induced inflammation by CEO at 1 mg/mL was found to be 86.17% ± 0.18%, whereas that of standard Ibuprofen at 1 mg/mL was found to be 91.09% ± 0.20% (Fig. 4). In the trypsin method, the percentage inhibition of trypsin-induced inflammation by CEO at 0.2 mg/mL was found to be 75.65% ± 2.28%, whereas that of standard Ibuprofen at the same concentration was found to be 75.67% ± 0.54% (Fig. 5).

## 4 Discussion

Main volatile components of the CEO were fatty alcohol and aldehyde such as 1-decanol (17.85%), decanal (11.04%), and *trans*-2-Dodecen-1-ol (7.87%) and monoterpene such as menthone (6.71%). Fatty alcohol and aldehyde lipid molecule are extensively found in several edible fruits and plants. *Cis*-2-decenal, *trans*-2-dodecenal, *trans*-2-dodecen-1-ol, 1-dodecanol, and *trans*-2-decenal in oil are the major compounds that provide a characteristic aroma to coriander leaves(Eyres et al., 2005).

The finding of our study is consistent with that of a study by Turkmen et al., in which 1-decanol (8.29%–16.16%), decanal (9.95%–16.53%), and *trans*-2-dodecen-1-ol (6.46%–12.65%) were identified as the major components of the Turkish Cilantro varieties collected from five different region of Turkey (Turkmen et al., 2016). In essential oil extracted from coriander leaves cultivated in Brazil, decanal (19.09%) was reported to be the main metabolite, followed by *trans*-2-decenal (17.54%), 2-decen-1-ol (12.33), and cyclodecane (12.15%) (Freires et al., 2014). Shahwar et al. (Shahwar et al., 2012) reported that essential oil obtained from the coriander leaf varieties grown in Pakistan comprises *trans*-2-decenal (32.23%), linalool (13.97%), and *trans*-2-dodecenal (7.5%), which is different from the composition of the essential oil obtained from coriander leaf cultivated in Saudi Arabia in terms of monoterpene hydrocarbon and oxygenated monoterpene contents (Shahwar et al., 2012). Cadwallader et al., studied the essential oil composition of the coriander leaves cultivated in America and reported that *trans*-2-decenal, *trans*-2-dodecenal, and *trans*-2-tetradecenal are the most abundant constituents in these essential oils(Cadwallader et al., 1999). Several other studies have reported that essential oil from coriander leaves typically contains 1-decanol (2–36%), *trans*-2-decenal (1–30%), linalool (0–26%), decanal (3%- 20%), *trans*-2-decen-1-ol (0–19%), *trans*-2-dodecen1-ol (0–18%), *trans*-2-tetradecenal (0–13%), and *trans*-2-undecenal (0–5%) as the major constituents (Rao et al., 2004);(Priyadarshi and Borse, 2014) ;(Satyal and Setzer, 2020) . The presence of unsaturated aldehydes such as *trans*-2-decenal, *trans*-2-tetradecenal, *trans*-2-decen-1-ol, and 1-decanol in coriander leaves is responsible for the aroma of leaves or other aerial parts of the plant(Eyres et al., 2005). Chung et al, reported that the essential oil of Korean *C. sativum* leaves mainly contain cyclododecanol (23.11%), tetradecanal, 2-dodecenal, 1-decanol, 13-tetradecenal, 1-dodecanol, dodecanal, and 1-undecanol, which clearly indicate a distinct chemotype of the coriander variety grown in Saudi Arabia (Chung et al., 2012). The chemical class characteristics of *C. sativum* and essential oils from different aerial parts of the plant were reported by Mandal and Mandal, and the variation in metabolites may be attributed to many factors such as temperature, climate, humidity, radiation, and harvesting period in the growing environment(Mandal and Mandal, 2015).

DPPH-induced FRS activity has been proposed to be the prime method for assessing the antioxidant potential of extracts or compounds. It is a rapid, simple, cheap, and widely used assay to evaluate the biological action of free radical scavengers as hydrogen donors(Huang et al., 2012). The reducing power (RA) of a compound is related to the presence of electron- or hydrogen-donating groups; free radicals are trapped or stabilised by an electron-donating group of the antioxidant(Zhang et al., 2015). As presented in Fig. 2B, the RA of CEO improved in a dose-dependent manner; the absorbance of the standard (ascorbic acid) was found to be 1.117 ± 0.01, 1.69 ± 0.11, and 1.94 ± 0.02 at 0.25, 1 and 5 mg/mL, respectively, whereas that of CEO was found to be 0.093 ± 0.01, 0.752 ± 0.01, and 1.217 ± 0.008 at 0.25, 1, and 5 mg/mL, respectively. The presence of decanol, 1-decanol, dodecanal, and other major chemicals in medicinal plants has been reported to be responsible for the FRS activity (Eom et al., 2017);(Abdullah et al., 2017) . Antioxidant properties of the CEO have also been reported earlier. According to Yildiz, the essential oil of Turkey coriander leaves mainly comprising *trans*-2-decenal (29.87%), in addition to linalool, *trans*-2-dodecenal, dodecanal, *trans*-2-undecenal, and other compound possesses antioxidative activities, as demonstrated through DPPH FRS and β-carotene bleaching assays (Yildiz, 2016). In addition, several other reports have confirmed the antioxidant properties of CEO(Wangensteen et al., 2004);(Iqbal et al., 2019) .

In the present study, *S. aureus*, *S. subtilis*, *E. coli*, *K. pneumoniae*, and *C. albicans* were selected for the antimicrobial assay because of their crucial role in several disorders.

A study assessed the cell-viable count over a time period to confirm the bactericidal and fungicidal activities of the CEO to typify the relationship between the concentration and exposure length(Freire et al., 2017). Essential oils obtained from the leaves of immature *C. sativum* L displayed an excellent MIC range for gram-positive bacteria, gram-negative bacteria, and fungus due to the presence of long chain fatty alcohols and aldehyde components (Delaquis et al., 2002). The inhibitory effect of *C. sativum* on *K. pneumoniae*; *P. aeruginosa*, *S. aureus*, and *E. coli* has been reported in the previous studies (Keskin and Toroglu, 2011);(Mandal and Mandal, 2015). In the present study, the essential oil of coriander leaves was found ineffective against *E. coli*. Antifungal activity and mechanism of action of leaf essential oil against *Candid*a spp. were reported in a study, and the effects were attributed to the essential oil binding with the ergosterol membrane, which increases the ionic permeability and causes membrane damage, leading to cell death (Freires et al., 2014). Several other studies have demonstrated the antimicrobial activities of CEO (Shahwar et al., 2012);(Sourmaghi et al., 2015) ;(Kačániová et al., 2020) .

Egg albumin activates several inflammatory mediators, and it is a convenient assay to assess the anti-inflammatory activity(Ullah et al., 2014). Trypsin induces activation and release of inflammatory mediators from human eosinophils through PAR2 (protease-activated receptor-2) and may be involved in inflammation and immunity (Miike et al., 2001). For the CEO, concentration-based denaturation inhibition of the inflammation induced by egg albumin was observed, and similar concentration-based inhibitions were noted in the proteinase inhibitory assay. The present study suggests that the anti-inflammatory activity of CEO is excellent and comparable to that of Ibuprofen. The anti-inflammatory activity of CEO has been attributed to the presence of fatty alcohol/aldehyde and several other components(Heidari et al., 2016);(Boudjema et al., 2018) . Other studies have also demonstrated the anti-inflammatory action of CEO(Priyadarshi and Borse, 2014);(Iqbal et al., 2019) .

## 5 Conclusion

Despite the extensive use of Cilantro in kitchen since a long time, modern science has recently started exploring its value as a scientific treasure for the treatment of different ailments. In the current work, CEO from the aerial and green parts of *C. sativum* was hydro distilled to explore its treasured properties. The CEO demonstrated antioxidant activities at extremely high concentrations; however, it demonstrated superior antimicrobial potential against *S. aureus*, *S. subtilis, K. pneumoniae*, and *C. albicans*. The CEO was also found to exhibit superior albumin denaturation (AD) and proteinase inhibition properties comparable to those of standard Ibuprofen. Overall, the results of the study suggest that the composition of CEO is responsible for its strong antimicrobial and anti-inflammatory (anti-denaturation and proteinase inhibitory) properties but weak antioxidant property.

## Declarations of interest:

None.
